# Construction Notes

**Published:** 1905-02-18

**Authors:** 


### CONSTRUCTION NOTES.

It is proposed to extend the Manchester Royal Eye Hospital. A cottage hospital is about to be erected for Crieff and its district at Pittenzie.

The Leeds General Infirmary is about to come into possession of the residuary estate of the late Mr. Christopher Weatherall, of Leeds. This amounts to over £116,000, and it will be devoted to the foundation and endowment of the Weatherall Accident Ward and to the surgical requirements of the infirmary.

